# Harnessing Digital Initiatives for Improved Health Outcomes in Diabetes Management: An Observational Patient Program

**DOI:** 10.7759/cureus.73093

**Published:** 2024-11-05

**Authors:** Bipin Sethi, Krishna Seshadri, Vaishali Deshmukh, Unnikrishnan AG, Manash Baruah, Sanjeev Phatak, Samit Ghosal, Sachin Chittawar, Khushboo Aggarwal, Bharath HS, Prashant Sada

**Affiliations:** 1 Endocrinology, CARE Hospitals & Transplant Centre and CARE Hospitals, Outpatient Centre, Hyderabad, IND; 2 Endocrinology, Apollo Speciality Hospital, Chennai, IND; 3 Endocrinology, Deenanath Mangeshkar Hospital, Pune, IND; 4 Endocrinology, Chellaram Hospital - Diabetes Care and Multispeciality, Pune, IND; 5 Endocrinology, Excelcare Hospital, Guwahati, IND; 6 Diabetes and Endocrinology, Vijayratna Diabetes Diagnosis & Treatment Centre, Ahmedabad, IND; 7 Endocrinology, Nightingale Hospital, Kolkata, IND; 8 Endocrinology, Harmony 360 Degree Diabetes Care, Bhopal, IND; 9 Medical Affairs, Zyla Health, Gurgaon, IND; 10 Medical Affairs, AstraZeneca Pharma India Ltd, Bangalore, IND

**Keywords:** diabetes mellitus, education and support intervention, mobile applications, mobile health, patient management system tool, personalized care

## Abstract

Introduction: Patients with diabetes have easy access to a wide range of digital applications that may help with self-management and lower barriers; however, robust evidence of their effectiveness remains somewhat elusive. Zyla is a medical artificial intelligence (AI)-based personalized care management app that assists the treating physician in improving the standard of patient care by offering the patients comprehensive and individualized care. This preliminary evaluation of data collected through the Zyla app aims to understand the impact of diabetes disease outcomes among patients subscribed to this app.

Methods: This was a retrospective, observational program conducted through the Zyla app in the calendar year 2020. The Zyla app's objective is to assist the treating physician in improving the standard of patient care by giving them the choice of assembling a personalized team (consisting of clinical nutritionists, physiotherapists, and counselors over a virtual platform) that can offer patients comprehensive and individualized care. Data on parameters like glycated hemoglobin (HbA1c), fasting blood sugar (FBS), post-prandial glucose (PPG), serum creatinine (SC), total cholesterol (TC), triglycerides (TG), high-density lipoprotein cholesterol (HDL-C) and low-density lipoprotein cholesterol (LDL-C) were collected through the Zyla app. Clinical outcomes assessed were the change from baseline to last reported levels of the mentioned parameters and are reported using descriptive analysis.

Results: The glycemic control parameters, HbA1c (change from baseline (CFB): -1.08), FBS (CFB: -15.93), and PPG levels (-18.42), were significantly lower (*P*<0.0001) at the last assessment compared with baseline. For the lipid profile, levels of TGs (*P*<0.0001) and TC (*P* = 0.0037) were significantly lower compared with baseline, while HDL-C levels were comparatively higher (CFB: 0.68) and LDL-C levels were lower (CFB:11.60), however non-significant. Serum creatinine was also lower compared to baseline (CFB: -0.25); however, the difference was not statistically significant.

Conclusions: A significant improvement in all glycemic parameters was seen with the use of the Zyla app along with numerical improvements in kidney function parameters and cholesterol status among patients. These preliminary findings warrant further rigorous studies to validate the impact of medical apps in the management of diabetics in India.

## Introduction

The world has recently seen a significant rise in healthcare digitization, transforming how healthcare services are delivered [1,2]. This global trend involves various technologies under digital health, promoting wellness, managing chronic diseases, and facilitating the collection, storage, and transmission of vital health information [2]. Social media, smartphones, mobile apps, wearable devices, cloud platforms, and real-world evidence studies have accelerated digital health adoption [3]. To address complicated health issues, the Indian government has launched several initiatives in the year 2019, such as the National Digital Health Mission, as part of the Digital India study [4]. India is the second-largest mobile phone user base in the world, and smartphone penetration may increase the efficacy of e-governance programs. Digital inclusion will transform primary care, specialty care, health promotion, and prevention in India, among other stages of healthcare delivery [4]. The International diabetes federation (IDF) estimated 537 million people living with diabetes and 6.7 million deaths due to diabetes, globally in 2021 [5]. India is the country with the second highest number (77.0 million) of people living with diabetes [5]. The numbers are expected to rise to 101.0 million in 2030 and to 134.2 million by 2045 [5]. The use of digital health technologies in the management of diabetes will not only facilitate informed therapeutic decisions but may also offer a promising avenue for self-management of diabetes in India. 

Traditional approaches to diabetes management have often been hindered by resource limitations and the inherent difficulties associated with delivering personalized care [6-8]. Patients with diabetes encounter several obstacles in their healthcare journey, including optimizing the use of existing therapies to maintain glycemic control, blood pressure, and lipid control while minimizing complications [8,9]. The TIGHT study, which was a large real-world study conducted in India reported approximately 76% of patients with diabetes had uncontrolled glycemia, and factors like obesity, hypertension, and diabetes duration >5 years (P<0.001) were significantly associated with uncontrolled glycemia [10]. Additionally, educating patients on effective self-management, enhancing treatment adherence, overcoming barriers to early diagnosis, and improving healthcare delivery to those with chronic conditions are paramount challenges that need to be addressed [8]. A study reported non-compliance by 24% of patients with diabetes with traditional approaches to diabetes management [11]. Digital management of chronic diseases like diabetes may help mitigate these barriers through constant follow-ups and reminders via the digital platform. Currently, numerous digital applications are readily available to patients with diabetes, which may aid in self-management and reduce the barriers, but the robust evidence of their effectiveness is limited [12]. Bridging this gap is essential to facilitate the widespread adoption of digital health applications in healthcare.

Driven by the widespread availability of smartphones and internet connectivity, medical app usage has increased significantly in India over the past decade. However, research data on the effectiveness of these app outcomes are limited, particularly in managing diabetes among the Indian population [13-15]. The aim of this preliminary analysis of data obtained from the Zyla app was to analyze the impact of the use of digital application on diabetes disease outcomes among Indians.

## Materials and methods

Program design

This was a retrospective, observational program conducted through the Zyla Virtual Care Clinic mobile-based application (Zyla app [16]) in the calendar year 2020.

Description of the Zyla app

**Figure 1:**
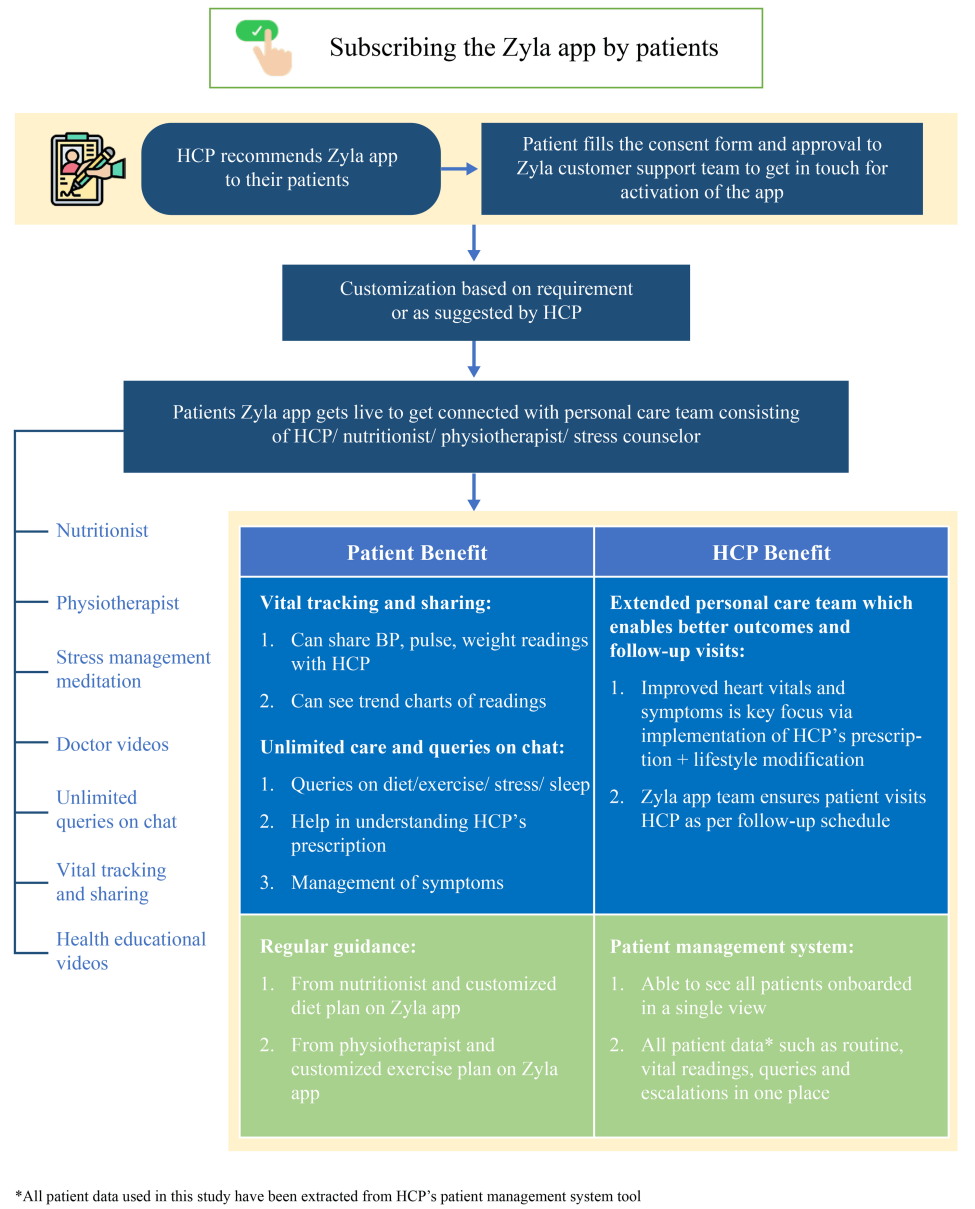
The Zyla app BP, blood pressure; HCP, healthcare professional

The goal of the Zyla app was to aid the treating physician enhance the quality of patient care, by providing an option to have a customized team that could provide personalized and holistic care to their patients (Figure 1). The Zyla app brought together an extended team of healthcare professionals (HCPs) comprising clinical nutritionists, physiotherapists, and counselors in a virtual set-up. This team could provide nutrition, exercise, and stress management counseling support to the patient through the mobile application in real time. Nutritional counseling to the patients was available in nine major regional languages including Hindi, English, Punjabi, Marathi, Urdu, Telugu, Kannada, Malayalam, Bengali, and Tamil. However, physiotherapists and psychologists used Hindi and English languages only for counseling.

There is an artificial intelligence (AI) enabled chatbot option also associated with the app. All the answers/responses given by the AI Chatbot were initially prepared by an in-house qualified team consisting of nutritionists, Physiotherapists, and Psychologists. Moreover, the AI Chatbot was supported by human assistance to ensure quality. All the responses provided by the AI Chatbot were reviewed by the in-house team for assurance and relevance perspective. Although overall the Zyla mobile application is available in English language only, in the Chatbot, patients could type in any regional language. The response from the chatbot could also be in the native language in such cases.

The Zyla app ensures that the patients are compliant with their treatment and stay connected with their treating physician in between clinic visits. The app provides not just reactive care (responding to patient data input), but also proactive care (reaching out to the patient every day based on health progress). Moreover, the app has a very strong follow-up mechanism to ensure adherence from patients. If patients were not responsive over chat, the host of the app communicated with the patients across other channels (SMS, WhatsApp, Email). Despite this, if there was no response, then the host of the app phoned the patient.

Additionally, the patient also receives personalized care and support to enable lifestyle modifications for improved healthcare outcomes. The Zyla app directs all medical queries to the treating physician, who can monitor the patient data on the Patient Management System provided by the Zyla app. The treating physician can see patient's data like routine reports, vital readings, queries, and escalation in one place and can always act based on the requirement. The app provides real-time responses to non-medical queries (queries related to diet, exercise, stress, etc.) between 7 am to 11 pm. The maximum response time was less than 5 minutes. Medical queries asked by the patient between 9:30 am and 7 pm were escalated to the HCP within 15 minutes of receiving. Queries received between 7 pm and 9:30 am were escalated by 10 am of the following day. In the current program, the Zyla app was recommended to the patients by their treating physicians and was available to the patients without any additional cost. Data of the patients who agreed to use the Zyla app in addition to their regular treatment were included in this program. Patient data were obtained from the patient management system tool of the Zyla app.

Assessments and outcomes

Data on parameters like glycated hemoglobin (HbA1c), fasting blood sugar (FBS), post-prandial glucose (PPG), serum creatinine (SC), total cholesterol (TC), triglycerides (TG), high-density lipoprotein cholesterol (HDL-C), and low-density lipoprotein cholesterol (LDL-C) were collected through the Zyla app. Clinical outcomes assessed were the change from baseline to the last reported levels of the parameters mentioned.

Based on the duration of usage of the Zyla app, all the outcomes were also assessed in subgroups of patients who used the Zyla app for less than six months, for 6 to 12 months, and more than 12 months.

Statistical analysis

A descriptive analysis was performed from the available data and changes from baseline data for each of the parameters were calculated. Continuous variables were presented in the form of means ± standard deviation (SD). Change from baseline (Final Visit - First Visit) was analyzed using a two-sided paired t-test at 0.05 level of significance. Statistical analyses were performed using SAS, version 9.4 (SAS Institute, Cary, NC).

Ethical consideration

Patient consent was recorded during the subscription of the app. No patient identification data was used for this publication. Any data collected is only used by the app for care delivery purposes; no personal data is passed on to any third party. Only in cases, when the patient requests discounted medicines, a callback is arranged from the app’s trusted partners. All data in transit, are encrypted, end to end using SSL/RSA 2048 bit certificates.

## Results

Data analysis is based on the data availability for each parameter from the Zyla app. For glycemic control parameters, data was available for 208 patients for HbA1c, 969 for FBS, and 714 for PPG. Data on the lipid profile was available from 100 patients for TC, 111 for TG, 105 for HDL-C, and 111 for LDL-C. We had 178 patients’ data for SC data analysis.

Outcomes

Glycemic control parameters including HbA1c, FBS, and PPG levels were significantly lower (P<0.0001) at the last assessment compared with baseline (Table 1). For the lipid profile, levels of TGs (P<0.0001) and TC (P=0.0037) were significantly lower compared with baseline, while HDL-C levels were comparatively higher (change from baseline (CFB): 0.68) and LDL-C levels were lower (CFB: -11.60), however non-significant (Table 1). Serum creatinine was also lower compared to baseline (CFB: -0.25); however, the difference was not statistically significant (Table 1).

**Table 1:** Mean change from baseline to last assessment for all the clinical parameters FBS, fasting blood sugar; HbA1c, glycated hemoglobin; HDL-C, high-density lipoprotein cholesterol; LDL-C, low-density lipoprotein cholesterol; PPG, post-prandial glucose; SC, Serum creatinine; TC, total cholesterol; TG, triglycerides

Clinical parameters	Overall duration months	Baseline	Last assessment	Change from baseline	P-value
HbA1c, % (n=208)	6.31 (5.921)	8.58 (2.088)	7.49 (1.501)	-1.08 (2.042)	<0.0001
FBS, mg/dL (n=969)	3.87 (4.315)	145.16 (51.208)	129.23 (38.937)	-15.93 (49.943)	<0.0001
PPG, mg/dL (n=714)	3.65 (4.192)	188.29 (72.942)	169.86 (60.547)	-18.42 (74.802)	<0.0001
SC, mg/dL (n=178)	6.48 (6.458)	1.42 (3.721)	1.16 (1.063)	-0.25 (3.68)	0.3509
TC, mg/dL (n=100)	6.57 (5.075)	195.79 (66.992)	176.70 (50.574)	-19.09 (64.256)	0.0037
TG, mg/dL (n=111)	6.74 (6.094)	231.06 (139.588)	186.19 (90.907)	-47.92 (114.06)	<0.0001
HDL-C, mg/dL (n=105)	6.93 (5.215)	41.32 (10.855)	42.00 (8.94)	0.68 (11.984)	0.5621
LDL-C, mg/dL (n=111)	6.45 (5.180)	118.29 (54.734)	106.68 (42.090)	-11.60 (53.169)	0.0234

Outcomes of the subgroup analysis

Zyla app users in the subgroup <6 months group (n=1,715) were higher for all clinical parameters compared to 6-12 months (n=549) and >12 months groups (n=232). Due to small sample sizes for the subgroups, inferential analysis was not feasible.

**Figure 2:**
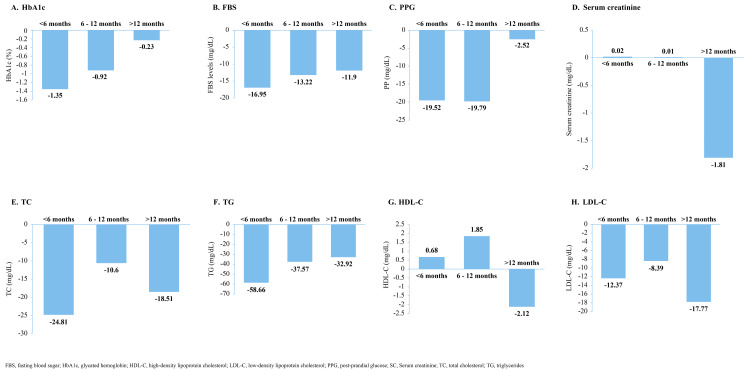
Mean change from baseline to last assessment by duration of Zyla app usage (subgroup analysis) FBS, fasting blood sugar; HbA1c, glycated hemoglobin; HDL-C, high-density lipoprotein cholesterol; LDL-C, low-density lipoprotein cholesterol; PPG, post-prandial glucose; SC, Serum creatinine; TC, total cholesterol; TG, triglycerides

HbA1c, FBS, and PPG were numerically lower at the last assessment compared to the baseline in all three subgroups (Table 2). Changes from baseline also showed a decreasing trend in all three groups (Figures 2A-2C). For HbA1c, the maximum change from baseline was observed in the <6 months group (-1.35) followed by 6-12 months group (-0.92) and the lowest change was observed in >12 months group (-0.23) (Figure 2A). For FBS (<6 months: -16.95; 6-12 months: -13.22; and >12 months: -11.9) and PPG (6-12 months: -19.79; <6 months: -19.52; and >12 months: -2.52), we observed similar trends. However, there was no consistent trend seen in the serum creatinine levels between the three subgroups. A slight increase in the levels of SC was observed in the <6 months and 6-12 months group, while a comparatively higher decrease in the SC level was observed in the >12 months group (Table 2). Consequently, change from baseline was also positive in the <6 months (0.02) and 6-12 months group (0.01) and negative in the >12 months group (-1.81) (Figure 2D).

**Table 2:** Outcomes in terms of the duration of Zyla app usage (subgroup analysis) FBS, fasting blood sugar; HbA1c, glycated hemoglobin; HDL-C, high-density lipoprotein cholesterol; LDL-C, low-density lipoprotein cholesterol; PPG, post-prandial glucose; SC, Serum creatinine; TC, total cholesterol; TG, triglycerides

	<6 months		6-12 months		>12 months	
	Baseline	Last assessment	Baseline	Last assessment	Baseline	Last assessment
HbA1c, %	n=114		n=72		n=22	
	8.90 (2.235)	7.54 (1.609)	8.32 (1.890)	7.39 (1.431)	7.80 (1.619)	7.56 (1.143)
FBS, mg/dL	n=731		n=164		n=74	
	146.47 (51.294)	129.51 (39.078)	143.10 (54.478)	129.88 (43.109)	136.96 (41.711)	125.05 (25.779)
PPG, mg/dL	n=549		n=117		n=48	
	189.42 (73.877)	169.90 (60.580)	184.82 (66.626)	165.03 (56.216)	183.81 (77.821)	181.29 (69.517)
SC, mg/dL	n=100		n=51		n=27	
	1.30 (1.268)	1.32 (1.360)	0.98 (0.413)	0.99 (0.417)	2.74 (9.253)	0.92 (0.314)
TC, mg/dL	n=52		n=34		n=14	
	199.33 (75.462)	174.52 (54.112)	193.23 (44.622)	182.63 (42.366)	188.90 (81.642)	170.39 (57.286)
TG, mg/dL	n=59		n=34		n=18	
	245.70 (157.232)	192.96 (95.303)	214.57 (120.383)	177.01 (90.925)	214.27 (109.614)	181.35 (78.005)
HDL-C, mg/dL	n=51		n=38		n=16	
	40.53 (8.278)	41.21 (7.400)	40.80 (9.003)	42.66 (10.439)	45.13 (19.249)	43.01 (9.930)
LDL-C, mg/dL	n=59		n=39		n=13	
	117.34 (59.882)	104.97 (43.756)	120.62 (46.001)	112.23 (38.937)	115.64 (58.309)	97.87 (44.569)

The lipid profile parameters (TC, HDL-C, LDL-C) also demonstrated inconsistent trends, except for TG levels, which decreased with an increase in duration (Table 2 and Figures 2E-2H).

## Discussion

In the current program, we investigated the effect of interventions pushed through the Zyla app in regulating glycemic control and lipid profile among the subscribers of the app. The preliminary results indicate significant improvements in HbA1c levels, FBS, and PPG levels. Additionally, there were improvements in the lipid profile, with lower TGs and TC levels, higher HDL-C levels, and lower LDL-C levels, although the latter change was not statistically significant. Serum creatinine levels also decreased over time, albeit not statistically significant. Overall, these findings suggest positive trends in glycemic control and lipid profiles among the app's subscribers. The benefits seen in terms of improvements in the laboratory parameters suggest that app-based interventions may motivate these patients to increase self-care and monitoring of the disease. 

Findings from our preliminary data analysis corroborate with the recommended guidelines and the current evidence from global studies. The American Diabetes Association guidelines recommend lifestyle interventions not only for the primary prevention of metabolic syndrome but also as a therapeutic component in the management of diabetes [17]. The effectiveness of lifestyle changes in terms of glycemic control for patients with T2DM has previously been demonstrated in several studies [18-20]. A meta-analysis reported a significant improvement in HbA1c (mean difference (MD) -0.63), FBG (MD -0.35), and BMI (MD -0.5) when lifestyle intervention, which included self-management via multiple education components like diet, physical activity, medication adherence, and smoking cessation were delivered by HCPs in hospital/clinic setting to patients with T2DM [18]. Another meta-analysis evaluating the effect of nutritional education, as a lifestyle intervention for patients with diabetes, reported reductions in body weight, HbA1c, FBS, and PPG levels [19]. A holistic approach that combines these elements may significantly enhance the well-being of individuals living with T2DM [21,22]. A randomized controlled trial was conducted to observe the effect of self-managed lifestyle treatment on glycemic control using a web-based digital tool in patients with T2DM. Users of the tool had reduced FBS and HbA1c accompanied by decreased body weight, fat mass, insulin resistance, increased muscle mass, reduced systolic blood pressure, and improved HDL-C [20]. Thus, digital health applications can provide an integrated platform incorporating these self-management elements for effective diabetes management. These apps can support patients in effective self-management, helping them monitor their progress and work towards achieving treatment goals. The convenience and accessibility of digital health tools make them valuable assets in the realm of diabetes care and prevention [20,23-25].

A recent multicenter, open-label trial assessing the impact of the digital health application reported significantly improved glycemic control and metabolic parameters in patients with T2DM [26]. Participants in this program received a digital lifestyle intervention via an app, which used available evidence from the fields of medical nutrition therapy, psychology, and behavioral intervention for patients with T2DM. HbA1c levels were observed three months before app use, at the start of usage, and three months after the start of use. An average reduction in HbA1c and FBS of 0.9% and 0.6 mmol/L (10.8 mg/dL; converted by multiplying the value by 18.018) was achieved. In addition, the app was also effective in significantly reducing body weight and BMI [26]. Along similar lines, in our program use of digital lifestyle intervention via the Zyla app resulted in significant and clinically meaningful reductions of 1.08% and 15.93 mg/dL in HbA1c and FBS, respectively. Another report from a large clinical trial conducted on the Indian population to assess the effectiveness of mobile phone messaging in the prevention of T2DM by lifestyle modification reported a lower cumulative incidence of developing T2DM (hazard ratio: 0·64, 95% CI 0·45-0·92; P=0·015) in the intervention group (18%, received mobile phone messages) compared with controls (27%, standard care)[27]. Participants in the intervention group received mobile phone message reminders for physical activity and diet plans. The frequency of messages was tailored according to five stages (pre-contemplation, contemplation, preparation, action, and maintenance) [27]. Patnaik et al. reported a significant reduction in HbA1c levels among Indian patients who used a mobile health app or website to get diabetes self-management education [28]. Two randomized controlled trials in the Asian population based on mobile health-based diabetes self-management education [29] and interactive platform [30] reported increased glycemic control and improved lipid profile compared to non-mhealth users. The current analysis reports an improvement in lipid profile, although insignificant, in the app users, which might be because patients with diabetes often have multiple comorbidities affecting lipid metabolism, such as hypertension, obesity, and metabolic syndrome, and therefore improvement in these conditions may require intensive management of these various arms [31]. 

Combining the summary of these reports, digital health technologies have indeed demonstrated promising results in enhancing diabetes care, enabling patients to take charge of their health conditions, as also seen in our preliminary analysis. The convenience and accessibility of these tools contribute significantly to improving overall healthcare experiences. 

In the current program, Zyla app users in the subgroup <6 months group were higher for all clinical parameters compared to 6-12 months and >12 months groups. The findings indicate variations in clinical parameters across different duration subgroups (<6 months, 6-12 months, and >12 months). Changes in HbA1c, FBS, and PPG levels showed varying trends, with the most substantial improvements observed in the <6 months group. Serum creatinine levels displayed inconsistent patterns, while lipid profile parameters showed mixed trends except for a decrease in TG levels with increasing duration. These inconsistent results reflect the usage of the app and further analysis could provide useful information to improve the patient engagement levels of these apps. A 3-month mobile health-based diabetes prevention trial conducted in India, reported non-significant reductions in FBS levels, with lower TGs and TC levels, higher HDL-C levels, and lower LDL-C levels among the participants using the mDiab app compared to the non-app users [32]. The mDiab app used in this program provided counseling to the patients via videos, short message service, and infographics through a smartphone application followed up weekly by health coach calls [32]. The Zyla app is different from the mDiab app which provides virtual care to patients through a team of healthcare professionals such as nutritionists and physiotherapists in addition to providing information through videos and infographics. The results reported in our program are however congruent to the mDiab trial.

The strengths of the current analysis include its real-world setting, which provides valuable insights into the utility and usage of digital intervention in everyday clinical practice among patients with diabetes in India. Additionally, the comprehensive nature of this digital intervention, which addresses multiple aspects of diabetes management, is a significant strength. Lastly, the app’s focus on multiple clinical outcomes offers a broad perspective on personalized holistic diabetes care. 

The exploratory and observational nature of our analysis, potential confounding factors such as the use of other health services or medications, patient demographics, adherence to the treatment, and baseline health status limit the interpretation of overall results. There was no comparator arm used in this program. We extracted only the data of patients who subscribed to the Zyla app and analyzed the CFB. Furthermore, the duration of evaluation for each parameter and each patient was non-uniform. However, the subgroup analysis conducted based on the duration of the app usage gave useful insights into the usage of the app and clinical outcomes. Due to small sample sizes for the subgroups, inferential analysis was not feasible as the power obtained was <80% in the statistical analysis for sample size calculation. A non-uniform sample size for each parameter was used which may affect the overall results and interpretation of the findings should be done cautiously. A larger sample size will help ensure the reliability and generalizability of the program's findings.

Furthermore, app-based limits are unsurmountable. First, due to privacy and security concerns, patient-level data such as demographics were not gathered. Such data cannot be used in reports according to the security policy of the app. Second, the educated population who can read and comprehend the HCP’s advice is primarily the target audience for the application. Thirdly, the app may be difficult to use for elderly users because they may not be tech-savvy, which might hamper the use of the app. Patient caregivers may offer help to such individuals to obtain the recommendations and guidance of the HCP. Lastly, potential biases might also have been introduced in the overall results due to the app's features, such as the AI chatbot, where personalized feedback varies between users, and multiple communication languages. 

Future research should be conducted utilizing the randomized controlled study design to validate these preliminary findings and assess the app’s effectiveness rigorously. Additionally, one should explore the app's impact in diverse populations and various healthcare settings to provide a comprehensive understanding of its utility and generalizability. These measures will help further establish the app's role in diabetes management and address any remaining uncertainties.

## Conclusions

In this manuscript, we aimed to analyze if the use of a digital application has an impact on disease outcomes and patient care. The Zyla app brought together a customized team of HCPs comprising clinical nutritionists, physiotherapists, and counselors who could provide personalized and holistic care. The Zyla app usage led to significant improvements across the diabetic parameters accessed (HbA1c, FBS, and PPG levels). Additionally, while there were improvements in kidney function (serum creatinine), and lipid status parameters (HDL-C and LDL-C levels), these changes were not statistically significant. These results were similar to other studies analyzing the effectiveness of digital healthcare initiatives to improve patient health outcomes. Since this is a preliminary, observatory analysis based on the Zyla app, further research in controlled settings is warranted to confirm and validate the findings of the program. 
